# Health surveillance at mass gatherings: experience report from the Strategic Health Surveillance Information Center in the city of Rio de Janeiro, 2024

**DOI:** 10.1590/S2237-96222025v34e20250212.en

**Published:** 2025-09-29

**Authors:** Isiyara Taverna Pimenta, Erika Fonseca Camargo Marsico, Alice Priscilla Miranda Souto, Maria Clara Henrique Moreira Geraldo, Léa de Freitas Amaral, Karoline Moreira Duffrayer, Caio Luiz Pereira Ribeiro, Gislani Mateus Oliveira Aguilar

**Affiliations:** ¹ Secretaria Municipal de Saúde do Rio de Janeiro, Rio de Janeiro, RJ, Brazil

**Keywords:** Public Health Surveillance, Mass Gatherings, Epidemiological Monitoring, Emergencies, Descriptive Study, Vigilancia en Salud Pública, Reuniones Masivas, Monitoreo Epidemiológico, Urgencias Medicas, Estudio descriptivo.

## Abstract

**Objective:**

To describe the health surveillance strategy implemented at mass gatherings by the Strategic Health Surveillance Information Center of the Rio de Janeiro Municipal Health Department, focusing on its activities at a music festival.

**Methods:**

This is an experience report. The music festival, which took place in 2024, was attended by 730,000 people. Before the event, operational preparations were carried out, and the local, national, and international epidemiological scenarios were monitored using digital detection tools. During the event, on-site actions were carried out, and active searches were conducted for cases of notifiable diseases/conditions and events of public health interest. During and after the event, local epidemiological surveillance was maintained to identify any potential changes associated with the festival.

**Results:**

Pre-event monitoring identified an increase in cases of whooping cough, outbreaks of diarrheal diseases, and the occurrence of measles, Oropouche virus, and avian influenza. During the event, 243 cases of notifiable diseases or conditions were reported, particularly influenza-like illness (42.0%) and exogenous intoxication (32.9%), as well as two outbreaks of exogenous intoxication and one outbreak of diarrheal disease. No changes were observed in the epidemiological scenario of the city of Rio de Janeiro following the event.

**Conclusion:**

The health surveillance response encompassed a comprehensive set of actions before, during, and after the event, enabling appropriate preparedness, early detection, and rapid response to potential public health emergencies. The lessons learned from this mass gathering will be incorporated into the planning and preparedness for future events.

## Resumen

**Objetivo:**

Describir la estrategia de vigilancia sanitaria implementada en eventos multitudinarios por el Centro de Información Estratégica en Vigilancia Sanitaria de la Secretaría Municipal de Salud de Río de Janeiro, destacando su actuación en un festival de música.

**Métodos:**

Este estudio es un relato de experiencia. El festival de música se celebró en 2024 y contó con la participación de 730 000 personas. Antes del evento, se llevaron a cabo preparativos operativos y se supervisó la situación epidemiológica local, nacional e internacional mediante herramientas de detección digital. Durante el evento, se llevaron a cabo acciones in situ y se buscaron activamente casos de enfermedades/afecciones de notificación obligatoria y eventos de interés para la salud pública. Durante y después del evento, se mantuvo el seguimiento epidemiológico local para identificar posibles cambios asociados al festival.

**Resultados:**

En el seguimiento previo al evento, se identificó un aumento de los casos de tos ferina, brotes de enfermedades diarreicas y la aparición de sarampión, oropouche y gripe aviar. Durante el evento, se registraron 243 casos de enfermedades/afecciones de notificación obligatoria, entre los que destacaron el síndrome gripal (42,0 %) y la intoxicación exógena (32,9 %), dos brotes de intoxicación exógena y uno de enfermedad diarreica. Tras el evento, no se observaron cambios en el panorama epidemiológico de la ciudad de Río de Janeiro.

**Conclusión:**

La actuación de la vigilancia sanitaria implicó un conjunto integrado de acciones antes, durante y después del evento, que permitieron una preparación adecuada, la detección precoz y la respuesta rápida a posibles emergencias de salud pública. Las lecciones aprendidas en el evento masivo se incorporarán a la planificación y preparación de eventos futuros.

**Ethical aspects**

This research respected ethical principles, having obtained the following approval data:

Research ethics committee: Secretaria Municipal de Saúde do Rio de Janeiro

Opinion number: 6,990,641

Approval date: 7/4/2024

Certificate of submission for ethical appraisal: 81464924.5.0000.5279

Informed consent record: Dismissed

## Introduction

Mass gatherings are collective activities of a cultural, social, religious, commercial, political, or sporting nature held over a defined period, which promote the movement and gathering of people of national and/or international origin in a given location ^1^. Despite having a significant economic impact on host cities by promoting economic growth and generating employment ^2^, mass gatherings can also pose challenges and risks to public health. 

The movement and concentration of a large number of people from different regions increase the risk of respiratory disease transmission ^3^, outbreaks of water- and foodborne diseases ^4^, accidents (such as cuts, sprains, and contusions) ^5^, exogenous intoxication due to illicit drug use ^6^, among other health conditions, which can burden healthcare facilities. Furthermore, depending on the location and type of event, both the audience and workers may be exposed to the effects of adverse weather conditions, such as high temperatures, which can cause sunburn, dehydration, heat exhaustion, and stroke ^7^. Such a scenario reinforces the importance of health surveillance actions at mass gatherings.

Health surveillance involves the collection, consolidation, and analysis of data, as well as the dissemination of health-related information, to support the planning and implementation of actions for health promotion, disease prevention and control, and mitigation of health conditions and risk factors. In Brazil, with the implementation of the National Health Surveillance Policy in 2018, guidelines, organizational approaches, and responsibilities were established for all levels of the Brazilian National Health System (SUS). One of the policy’s guidelines applicable to the context of mass gatherings refers to risk management through strategies for identifying, planning, intervening, communicating, and monitoring risks, diseases, and health conditions ^8^. 

Given the potential public health impacts of mass gatherings, the municipal health surveillance system in Rio de Janeiro is tasked with monitoring such events. This role is particularly relevant considering the city has hosted major national and international events, such as the Military World Games, Rio+20, World Youth Day, the FIFA World Cup, the Olympic and Paralympic Games, the Conmebol Copa América, and the G20, as well as recurring events such as music festivals, New Year’s Eve celebrations, and Carnival. 

The objective of this study was to describe the health surveillance strategy implemented by the Health Surveillance Information Center of the city of Rio de Janeiro (CIEVS-Rio) during mass gatherings, with a focus on the experience at a music festival. 

## Methods 

This is an experience report. CIEVS-Rio is part of the Health Surveillance Superintendency, the municipal health surveillance management body of the city of Rio de Janeiro. At the time this study was conducted, the team consisted of a multidisciplinary group with additional training in public health and/or epidemiology, comprising four nurses, one nutritionist, one biomedical scientist, one pharmacist, and one nursing technician. This team coordinates surveillance, alert, and response actions related to public health events and emergencies in the city of Rio de Janeiro, as well as the monitoring of mass gatherings ^9^. Public health events refer to the occurrence of outbreaks or epidemics, diseases of unknown etiology, and changes in the clinical and epidemiological pattern of known diseases. Public health emergencies, in turn, are situations that require the immediate implementation of prevention and control measures to contain risks, harms, and health impacts ^10^.

The music festival took place over seven days in September 2024 in Barra da Tijuca, Rio de Janeiro, with gates opening at 2 p.m. and closing at 4 a.m. The venue featured food courts, hydration stations, seven stages where concerts were held, and seven health stations strategically located near each stage to serve both the audience and workers. The estimated attendance throughout the event was 730,000 people, approximately 100,000 per day. 

Starting in May 2024, following the Madonna concert that drew over one million people to Copacabana Beach, CIEVS-Rio began to structure its response to mass gatherings in three stages: pre-event, during the event, and post-event (Figure 1). This approach resulted from extensive technical discussions among CIEVS-Rio team members, who recognized the need to expand their activities beyond the previous focus on the “during-event” stage. The new structure enabled better preparedness of health services and health surveillance based on the epidemiological scenario identified during the pre-event phase. It allowed for the evaluation of the mass gathering’s impact on public health in the post-event phase.

**﻿Figure 1.**
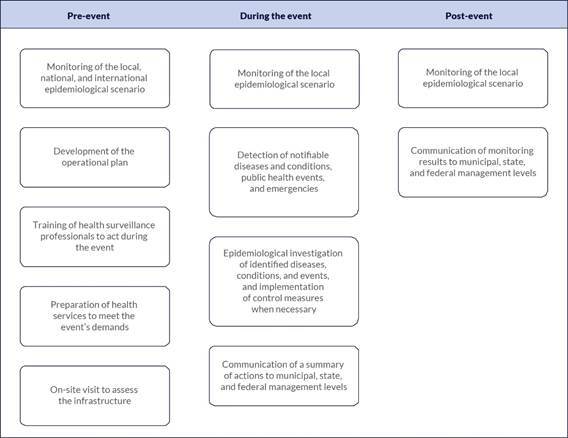
Health surveillance strategy implemented during mass gatherings by the Strategic Health Surveillance Information Center. City of Rio de Janeiro, September 2024

During the pre-event phase, continuous monitoring of the local, national, and international epidemiological scenarios was conducted using digital detection strategies based on event-based surveillance. Event-based surveillance involves the collection, monitoring, assessment, and interpretation of unstructured data related to potential public health risks. Data sources in this model are diverse and may include community rumors and news from newspapers, magazines, and websites, thereby complementing other surveillance strategies ^11^. 

The tool used by CIEVS-Rio was developed to collect data on notifiable diseases and conditions, public health events, and emergencies from official sources (such as websites and technical documents from InfoGripe, the Brazilian Ministry of Health, the World Health Organization, the Centers for Disease Control and Prevention, the United Nations, and the Epidemic Intelligence from Open Sources Initiative) and unofficial sources (any website reporting news on the aforementioned topics). The purpose of this tool was to enable early warnings of public health threats. 

**Figure 2.**
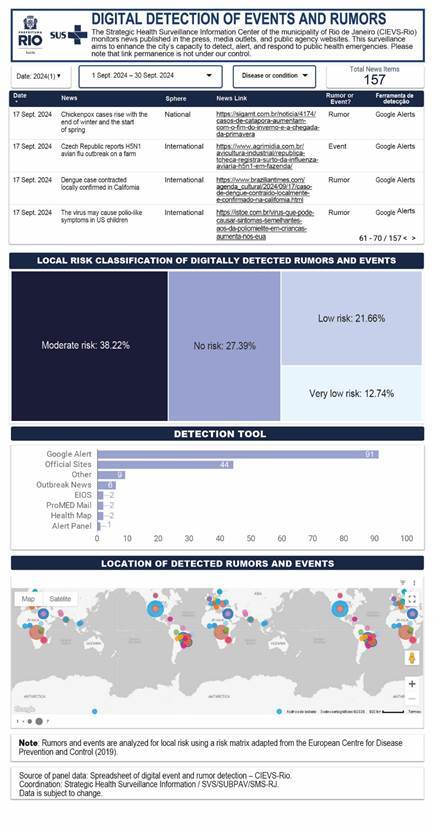
Main screen of the event-based surveillance dashboard showing the results of local, national, and international epidemiological scenario monitoring. City of Rio de Janeiro, September 2024

The data collected were routinely systematized, analyzed based on local risk (i.e., for the city of Rio de Janeiro), organized, and recorded on a dedicated dashboard developed by the CIEVS-Rio team (Figure 2). A local risk analysis was conducted using a risk matrix adapted from the operational tool of the European Centre for Disease Prevention and Control (12). This matrix was used to assess the likelihood that an event detected through event-based surveillance could occur in the city of Rio de Janeiro. It also allowed for the evaluation of the potential consequences such an event could have for the health of the local population. 

The purpose of local epidemiological monitoring was to track the distribution of diseases and health conditions and to identify patterns of occurrence. At the national and international levels, the focus was on detecting diseases with the potential to constitute public health emergencies or to cause changes in epidemiological patterns in the city of Rio de Janeiro due to population movement. The duration of monitoring was determined based on the incubation and transmissibility periods of the diseases identified in the assessed scenario. 

At the same time, focus groups were conducted and coordinated by the Rio de Janeiro Municipal Government through the Rio de Janeiro Municipal Tourism Company. They included the health (care and surveillance), transportation, security, ports and airports sectors, and the organizing company, among others, to plan actions during the event. As a result of these in-person meetings, each sector developed an operational plan, a technical document detailing the activities to be carried out, roles and responsibilities, and operational workflows. 

Following the development of the Health Surveillance Operational Plan, the CIEVS-Rio team conducted an on-site visit to assess the infrastructure and subsequently trained surveillance professionals through online or in-person meetings, where the Operacional Plan was presented and discussed in detail. The team also designated a referral health unit for patients meeting the criteria for laboratory investigation of notifiable diseases, thus preparing health services for the demands associated with the event.

During the mass gathering, continuous monitoring of environmental risks and active case searches for notifiable diseases ^13^, outbreaks, and other public health events of interest were conducted. The active case search consisted of reviewing medical reports from health stations at the event site, as there was no electronic information system in place to facilitate real-time identification of potential public health events. Epidemiological investigation and prevention and control measures were implemented when necessary. Additionally, cases of public health importance that required hospital referral were closely monitored. The information collected was consolidated daily and forwarded to the local health surveillance unit responsible for the event area, ensuring continuity of investigations and proper reporting in the Health Information System. The CIEVS-Rio team operating during the music festival consisted of seven health professionals: four nurses, one nutritionist, one pharmacist, and one biomedical scientist. Resources used included an institutional mobile phone, laptop, internet-connected tablet, printed notification forms, clipboards, and office supplies. The Integrated Joint Health Operations Center was shared with teams from the Municipal Institute of Health Surveillance, Zoonosis Surveillance, and Agricultural Inspection, as well as the General Coordination Office of the Regulatory Complex, facilitating intersectoral discussions.

Epidemiological monitoring of the city was maintained both during and after the mass gathering to detect any potential changes associated with it. It included an analysis of Sentinel Surveillance data, Health Information Systems, and public healthcare unit reports, which were published on the Epidemiological Intelligence Center dashboards ^14^. Sentinel Surveillance is a model in which laboratory samples are collected weekly at strategic health facilities to monitor arboviruses, influenza-like illnesses, acute diarrheal diseases, and conjunctivitis, aiming to detect their causative agents early ^9^. The consolidated results of the post-event monitoring were submitted weekly via technical reports to state and federal health authorities.

## Results 

In the pre-event phase, daily analysis of the epidemiological scenario over the previous four weeks identified an increase in whooping cough cases at the local, national, and international levels; one imported case of measles at the national level; outbreaks of acute diarrheal disease at the national level; cases of Oropouche at the national (extra-Amazonian region) and international levels; and human cases of avian influenza after occupational exposure at the international level. Based on this data, a quick guide was developed with guidelines on measles, chickenpox, mpox, arboviruses, and whooping cough to assist health center workers at the event in identifying cases and differentiating between cases of exanthematous diseases and mpox. A referral flow was also established for suspected cases of these diseases to the emergency health unit closest to the event site in order to facilitate the collection of samples for laboratory confirmation.

On each day of the event, the seven health posts were initially visited to introduce the surveillance team and provide guidance on notifiable diseases and conditions, as well as events and public health emergencies. Subsequently, rounds were conducted every three hours at the health posts to assess the service reports and actively search for cases of notifiable diseases, conditions, and events. A total of 135 rounds were carried out. At the end of each round, data on care, notifications, and referrals were forwarded to the municipal health surveillance management. At the end of each day, the consolidated information was forwarded to the local health surveillance, the Rio de Janeiro State Health Department, and the Ministry of Health. 

Of the total visits registered at the event’s health posts (n=8,835), 2.7% (n=243) were related to notifiable diseases and conditions. Among these, the majority were cases of influenza-like illness (42.0%) and exogenous intoxication (32.9%). Three outbreaks were identified and reported: one of acute diarrheal disease and two of exogenous intoxication caused by pepper spray. These cases facilitated epidemiological investigation and the implementation of on-site control measures (Table 1). 

**Table 1:** Absolute (n) and relative (%) frequencies of diseases, conditions, and public health events reported during the music festival. City of Rio de Janeiro, September 2024 (n=243)

**Diseases/conditions/events**	**n (%)**
Influenza-like illness	102 ( 42.0)
Exogenous intoxication	80 ( 32.9)
Work-related injury	45 ( 18.5)
Venomous animal bite/sting	9 ( 3.7)
Interpersonal violence	2 ( 0.8)
Dengue	2 ( 0.8)
Exogenous intoxication outbreak	2 ( 0.8)
Acute diarrheal disease outbreak	1 ( 0.4)

In the post-event phase, the local epidemiological scenario was monitored during the four weeks following the event. No changes were identified in the epidemiological profile of the city of Rio de Janeiro. The results were submitted weekly to the State Health Department of Rio de Janeiro and the Brazilian Ministry of Health via technical reports. 

To evaluate the performance during the mass gathering, a technical meeting was held with the CIEVS-Rio team and the Health Surveillance Superintendency to reflect on the method used and discuss areas for improvement. As an outcome of this meeting, the following strategies were defined for implementation in future events: 

Monitoring of heat levels in the city of Rio de Janeiro before and during mass gatherings: Given the current context of climate change and the increasing frequency of extreme heat events in the municipality, the city administration has established a classification system for heat levels based on metrics that combine temperature, relative humidity, and exposure time ^15^. Monitoring these levels will enable the planning and implementation of strategies to mitigate the adverse health effects of heat on the population and workers, especially during mass gatherings.

Monitoring of event-related visits within the Urgent and Emergency Care Network: Data from the electronic medical records of the Urgent and Emergency Care Network will be analyzed through the dashboards of the Epidemiological Intelligence Center (14). The search will be conducted using specific descriptors related to the event, which will allow for the identification of cases that sought care at external health facilities.

## Discussion 

The objective of this study was to describe the health surveillance strategy implemented by CIEVS-Rio during mass gatherings held in the city of Rio de Janeiro, with an emphasis on the experience during a music festival. The strategy involved active surveillance and event-based surveillance activities. The early identification of epidemiological risks, such as the rise in whooping cough and measles cases, enabled the development of educational materials and the organization of referral protocols for suspected cases identified at the event to be directed to municipal health units. The active surveillance carried out during the event allowed for the timely detection of notifiable diseases and conditions, as well as the immediate identification and response to outbreaks. Following the event, continuous monitoring of the epidemiological scenario revealed no changes in the pattern of disease occurrence in the city. The experience provided lessons that will be incorporated into the preparation of future events.

This experience report highlights some limitations that should be considered. As it describes a health surveillance strategy developed for a specific urban context, the methods employed may not be replicable in other locations, particularly those with less robust health infrastructure or different socio-environmental characteristics. The analysis of health data focused on visits to health posts during the event, which may not fully reflect all related cases, given that both the audience and workers may seek care at external health facilities.

Planning and response to the spread of communicable diseases and the occurrence of events and public health emergencies resulting from the intense national and international flow of people were also discussed in other publications ^3^ ^,^ ^4^ ^,^ ^5^ ^,^ ^6^ ^,^ ^16^ ^,^ ^17^ ^,^ ^20^. Similarly to the health surveillance strategy described in this study, those publications highlight actions for the prevention and mitigation of public health risks during mass gatherings, particularly epidemiological monitoring, the provision of on-site health services, and the development of rapid response protocols ^17^ ^,^ ^20^. However, to the best of our knowledge, no studies have been identified that systematize health surveillance actions in mass gatherings by considering the pre-event, during-event, and post-event stages, which makes the approach described here innovative. 

Although most literature on mass gatherings focuses on communicable diseases, other factors, such as risks associated with accidents, extreme weather events, and inadequate infrastructure, can also result in fatalities and disasters during large-scale events and should, therefore, be considered ^18^. The assessment of local infrastructure and environmental risks conducted by the health surveillance of the city of Rio de Janeiro team in the pre-event phase aligns with these recommendations. Monitoring weather conditions and their health impacts on event attendees is a strategy that will be implemented in future mass gatherings as one of the “lessons learned” from the music festival experience described in this report. 

High population density, adverse environmental conditions, and the potential for the transmission of communicable diseases require strategic planning and coordinated intersectoral actions. In this context, implementing sensitive and responsive surveillance systems that can detect early health warning signals and enable rapid and integrated responses becomes increasingly relevant ^5^ ^,^ ^17^. Introducing new technologies into surveillance systems has significantly contributed to this scenario by enabling real-time data collection and effective communication among teams, which in turn allows for more timely and accurate interventions. Although the city of Rio de Janeiro has not yet developed a dedicated information system for epidemiological monitoring during mass gatherings, the active search for cases of notifiable diseases and conditions has fulfilled this role. Furthermore, the future use of data from the Epidemiological Intelligence Center’s dashboards will enable the monitoring of relevant diseases and conditions beyond the physical boundaries of the event site.

The primary notifiable diseases and conditions reported during the event were exogenous intoxication, influenza-like illness, and work-related injuries. Alcohol and drug use stood out as major reasons for seeking health care at mass gatherings, leading to a significant increase in cases of intoxication, in addition to trauma and accident-related injuries ^17^ ^,^ ^18^. These findings are consistent with the results of this study, as most exogenous intoxication cases were linked to excessive alcohol and drug use. 

Reports of influenza-like illness may be partly attributed to the seasonality of respiratory viruses in Brazil, such as influenza and respiratory syncytial virus, which circulate more intensely during certain times of the year, especially in autumn and winter ^19^. This seasonality contributes to the higher incidence of respiratory diseases, particularly in crowded environments such as music festivals, where proximity among individuals facilitates the transmission of respiratory viruses.

CIEVS-Rio’s actions during mass gatherings involve a comprehensive set of measures before, during, and after the event. These are essential for the early identification of risks and the rapid response to situations that may constitute public health emergencies. Such measures aim to mitigate risks and ensure health safety in contexts of large gatherings, where the diversity of participants’ origins can intensify epidemiological challenges. At the music festival, the implemented actions enabled appropriate preparedness, early detection, and prompt response to potential public health emergencies. The lessons learned will support the planning of future events. 

Sharing CIEVS-Rio’s strategies and experiences may contribute to the continuous improvement of health surveillance practices at mass gatherings, strengthening the response capacity of different management levels within the Brazilian National Health System that face similar health challenges. The exchange of experiences between municipalities and the strengthening of surveillance networks are crucial strategies for the ongoing improvement of health surveillance practices at mass gatherings. These actions not only enhance local response capacity but also significantly contribute to the training of technical teams, promoting more efficient and integrated responses.
