# Supplementary material for: Health surveillance at mass gatherings: experience report from the Strategic Health Surveillance Information Center in the city of Rio de Janeiro, 2024
Source: Epidemiol Serv Saude. 2025 Sep 29;34:e20250212. doi: 10.1590/S2237-96222025v34e20250212.en (PMC12479030; doi:10.1590/S2237-96222025v34e20250212.en)
Supplement: Translation

# Translation: Vigilância em saúde em eventos de massa: relato da experiência do Centro de Informações Estratégicas em Vigilância em Saúde da cidade do Rio de Janeiro, 2024

**DOI:** 10.1590/S2237-96222025v34e20250212.pt

**Authors:** Isiyara Taverna Pimenta, Erika Fonseca Camargo Marsico, Alice Priscilla Miranda Souto, Maria Clara Henrique Moreira Geraldo, Léa de Freitas Amaral, Karoline Moreira Duffrayer, Caio Luiz Pereira Ribeiro, Gislani Mateus Oliveira Aguilar

**Affiliations:** ¹ Secretaria Municipal de Saúde do Rio de Janeiro, Rio de Janeiro, RJ, Brasil

**Keywords:** Vigilância em Saúde Pública, Eventos de Massa, Monitoramento Epidemiológico, Emergência, Estudo Descritivo

## Abstract

**Objetivo:**

Descrever a estratégia de vigilância em saúde implementada em eventos de massa pelo Centro de Informações Estratégicas em Vigilância em Saúde da Secretaria Municipal de Saúde do Rio de Janeiro, destacando a atuação em um festival de música.

**Métodos:**

Este estudo é um relato de experiência. O festival de música ocorreu em 2024 e teve a participação de 730 mil pessoas. No pré-evento, foram realizadas preparações operacionais e o monitoramento do cenário epidemiológico local, nacional e internacional por meio de ferramentas de detecção digital. Durante o evento, foram conduzidas ações in loco e busca ativa de casos de doenças/agravos de notificação compulsória e eventos de interesse para a saúde pública. Durante e após o evento, foi mantido o monitoramento epidemiológico local para identificar possíveis alterações associadas ao festival.

**Resultados:**

No monitoramento pré-evento, identificou-se o aumento de casos de coqueluche, surtos de doença diarreica e a ocorrência de sarampo, oropouche e gripe aviária. Durante o evento, foram registrados 243 casos de doenças/agravos de notificação compulsória, com destaque para síndrome gripal (42,0%) e intoxicação exógena (32,9%), dois surtos de intoxicação exógena e um de doença diarreica. No pós-evento, não foram observadas alterações no cenário epidemiológico da cidade do Rio de Janeiro.

**Conclusão:**

A atuação da vigilância em saúde envolveu um conjunto integrado de ações antes, durante e após o evento, que oportunizaram a preparação adequada, detecção precoce e resposta rápida a potenciais emergências em saúde pública. Os aprendizados adquiridos no evento de massa serão incorporados ao planejamento e à preparação de eventos futuros.

**Aspectos éticos**

Esta pesquisa respeitou os princípios éticos, obtendo os seguintes dados de aprovação:

Comitê de ética em pesquisa: Secretaria Municipal de Saúde do Rio de Janeiro

Número do parecer: 6.990.641

Data de aprovação: 7/4/2024

Certificado de apresentação de apreciação ética: 81464924.5.0000.5279

Registro do consentimento livre e esclarecido: Não se aplica, pois são utilizados dados secundários.

## Introdução

Eventos de massa são atividades coletivas de natureza cultural, social, religiosa, comercial, política ou esportiva, realizadas em período estabelecido, que promovem a circulação e aglomeração de pessoas, de origem nacional e/ou internacional, em determinada localidade ^1^. Apesar de apresentarem impacto econômico significativo para as cidades em que são realizados, promovendo crescimento econômico e gerando empregos ^2^, os eventos de massa também podem representar desafios e riscos para a saúde pública. 

O deslocamento e a concentração de grande número de pessoas de diferentes origens aumentam o risco de transmissão de doenças respiratórias ^3^, surtos de doenças transmitidas por água e alimentos ^4^, acidentes (como cortes, torções e contusões) ^5^, intoxicação exógena por uso de drogas ilícitas ^6^, entre outros agravos, o que pode sobrecarregar as estruturas de saúde locais. Além disso, dependendo do local e tipo de evento, tanto o público quanto os trabalhadores podem estar suscetíveis aos efeitos de condições climáticas adversas, como temperaturas elevadas, que podem causar queimaduras na pele, desidratação, exaustão e acidente vascular cerebral ^7^. Esse cenário reforça a importância de ações de vigilância em saúde em eventos de massa.

A vigilância em saúde envolve a coleta, consolidação e análise de dados, bem como a disseminação de informações relacionadas à saúde, com a finalidade de subsidiar o planejamento e a implementação de ações de promoção da saúde, prevenção e controle de doenças, agravos e fatores de risco. No Brasil, com a implementação da Política Nacional de Vigilância em Saúde em 2018, foram instituídas diretrizes, abordagens organizacionais e responsabilidades para todas as instâncias do Sistema Único de Saúde. Uma das diretrizes aplicável ao contexto de eventos de massa se refere à atuação na gestão de riscos por meio de estratégias de identificação, planejamento, intervenção, comunicação e monitoramento de riscos, doenças e agravos ^8^. 

Considerando os potenciais impactos dos eventos de massa para a saúde pública, a vigilância em saúde do município do Rio de Janeiro possui como atribuição o monitoramento desses eventos. Essa atuação é especialmente relevante, uma vez que a cidade já sediou importantes encontros nacionais e internacionais, como os Jogos Mundiais Militares, a Rio+20, a Jornada Mundial da Juventude, o Campeonato Mundial de Futebol da Fifa, os Jogos Olímpicos e Paralímpicos, a Copa América Conmebol e o G20, e organiza eventos recorrentes como festivais de música, Réveillon e Carnaval. 

O objetivo deste estudo foi descrever a estratégia de vigilância em saúde implementada pelo Centro de Informações Estratégicas em Vigilância em Saúde da cidade do Rio de Janeiro (CIEVS-Rio) em eventos de massa, destacando a experiência em um festival de música. 

## Métodos 

Este estudo é um relato de experiência. O CIEVS-Rio está alocado na Superintendência de Vigilância em Saúde, instância de gestão municipal da vigilância em saúde da cidade do Rio de Janeiro, era constituído à época da realização deste estudo por equipe multiprofissional, com formação complementar em saúde coletiva e/ou epidemiologia, que incluía quatro enfermeiros, uma nutricionista, uma biomédica, uma farmacêutica e uma técnica de enfermagem. Essa equipe coordena as ações de vigilância, alerta e resposta a eventos e emergências em saúde pública na cidade do Rio de Janeiro, além de atuar no monitoramento de eventos de massa ^9^. Entende-se por eventos de saúde pública a ocorrência de surtos ou epidemias, doenças de etiologia desconhecida e alterações no padrão clínico epidemiológico das doenças conhecidas. Por sua vez, as emergências em saúde pública são situações que demandam o emprego imediato de medidas de prevenção e controle para conter riscos, danos e agravos à saúde pública ^10^.

O festival de música ocorreu durante sete dias de setembro de 2024, na Barra da Tijuca, na cidade do Rio de Janeiro, com a abertura dos portões às 14h e o encerramento às 4h. O local contava com áreas de alimentação, postos de hidratação, sete palcos, onde ocorreram os shows de música, e sete postos de saúde, estrategicamente posicionados próximos a cada palco, para atender ao público e aos trabalhadores. O público estimado durante todo o evento foi de 730 mil pessoas, aproximadamente 100 mil por dia. 

A partir de maio de 2024, com a realização do show da Madonna, que reuniu mais de 1 milhão de pessoas na praia de Copacabana, a atuação do CIEVS-Rio nos eventos de massa passou a ser estruturada em três etapas: pré-evento, durante o evento e pós-evento (Figura 1). Essa abordagem foi resultante de intensas discussões técnicas entre os membros da equipe do CIEVS-Rio, que reconheceram a necessidade de ampliar a atuação em eventos de massa, anteriormente concentrada na etapa “durante o evento”. A nova estrutura possibilitou a preparação mais adequada dos serviços de saúde e da vigilância em saúde diante do cenário epidemiológico identificado na fase pré-evento e a avaliação dos impactos do evento de massa na saúde pública carioca no pós-evento.

**Figura 1:** Estratégia de vigilância em saúde implementada em eventos de massa pelo Centro de Informações Estratégicas em Vigilância em Saúde. Município do Rio de Janeiro, setembro de 2024

No pré-evento, foi realizado o monitoramento contínuo do cenário epidemiológico local, nacional e internacional por meio de estratégias de detecção digital da vigilância baseada em eventos. A vigilância baseada em eventos consiste na coleta, no monitoramento, na avaliação e na interpretação de dados não estruturados sobre potenciais riscos à saúde pública. As fontes de dados desse modelo de vigilância são diversas, podendo incluir rumores de membros da comunidade, notícias veiculadas em jornais, revistas e sites, complementando, assim, outras estratégias de vigilância ^11^. 

A ferramenta utilizada pelo CIEVS-Rio foi desenvolvida para coletar dados sobre doenças e agravos de notificação compulsória, eventos e emergências de saúde pública em fontes oficiais (sites e documentos técnicos do InfoGripe, do Ministério da Saúde, da Organização Mundial da Saúde, dos Centros de Controle e Prevenção de Doenças, da Organização das Nações Unidas, da Epidemic Intelligence from Open Sources Initiative, entre outros) e não oficiais (qualquer site que veiculava notícias sobre os assuntos supracitados). Essa ferramenta possui como objetivo promover o alerta precoce de ameaças à saúde pública. 

Os dados coletados foram sistematizados rotineiramente, analisados quanto ao risco local (para a cidade do Rio de Janeiro), organizados e registrados em painel próprio desenvolvido pela equipe do CIEVS-Rio (Figura 2). A análise do risco local foi realizada por meio de matriz de risco adaptada da ferramenta operacional do European Centre for Disease Control and Prevention ^12^, por meio da qual se avaliava a probabilidade de o evento capturado na vigilância baseada em eventos ocorrer na cidade do Rio de Janeiro e as consequências que ele traria para a população carioca caso ocorresse. 

O monitoramento do cenário epidemiológico local objetivou acompanhar a distribuição de doenças e agravos e identificar padrões de ocorrência. O monitoramento dos cenários nacional e internacional concentraram-se na detecção de doenças com potencial para configurar emergências em saúde pública ou alterações nos padrões epidemiológicos na cidade do Rio de Janeiro decorrentes do trânsito de pessoas. A duração do monitoramento foi determinada considerando o período de incubação e transmissibilidade das doenças identificadas no cenário analisado. 

Paralelamente foram conduzidos grupos focais, coordenados pela Prefeitura da Cidade do Rio de Janeiro, por meio da Empresa de Turismo do Município do Rio de Janeiro, que envolveram os setores de saúde (assistência e vigilância), transporte, segurança, portos e aeroportos, empresa organizadora, entre outros, para planejar a atuação durante o evento. Como resultado desses encontros presenciais, cada setor elaborou um plano operacional, documento técnico que detalhava as atividades a serem executadas, as atribuições e as responsabilidades profissionais, além dos fluxos operacionais. 

Após a elaboração do Plano Operacional de Vigilância em Saúde, a equipe do CIEVS-Rio realizou uma visita ao local do evento para avaliar a infraestrutura e, em seguida, treinou os profissionais da vigilância, por meio de reuniões (on-line ou presenciais) nas quais o plano operacional foi apresentado e discutido ponto a ponto. A equipe também definiu uma unidade de saúde de referência para o encaminhamento de pacientes que atendessem aos critérios para investigação laboratorial de doenças de notificação compulsória, preparando os serviços de saúde para as demandas relacionadas ao evento.

Durante o evento de massa, foi realizado o monitoramento contínuo dos riscos ambientais e a busca ativa para a detecção de casos de notificação compulsória ^(^ ^13^, surtos e outros eventos de interesse para a saúde pública. A busca ativa consistiu na avaliação dos boletins de atendimento dos postos de saúde disponíveis no local do evento, devido à ausência de sistema de informação eletrônico que possibilitasse a identificação, em tempo real, de potenciais eventos em saúde pública. A investigação epidemiológica e as medidas de prevenção e controle foram aplicadas conforme necessário. Além disso, foram acompanhados os casos de importância para a saúde pública que demandaram remoção hospitalar. 

**Figura 2:** Tela principal do painel da vigilância baseada em eventos com os resultados do monitoramento dos cenários epidemiológicos local, nacional e internacional. Município do Rio de Janeiro, setembro de 2024

As informações coletadas foram consolidadas diariamente e repassadas à vigilância em saúde local, responsável pelo território do evento, para a continuidade das investigações e notificação nos Sistemas de Informação em Saúde. A equipe do CIEVS-Rio que atuou durante o festival de música foi constituída por sete profissionais de saúde: quatro enfermeiros, uma nutricionista, uma farmacêutica e uma biomédica. Os recursos utilizados incluíram telefone celular institucional, notebook, tablet com acesso à internet, fichas de notificação impressas, pranchetas e material de escritório. O Centro Integrado de Operações Conjuntas da Saúde foi compartilhado com as equipes do Instituto Municipal de Vigilância Sanitária, Vigilância de Zoonoses e Inspeção Agropecuária e com a Coordenadoria Geral do Complexo Regulador, o que possibilitou discussões intersetoriais.

O monitoramento epidemiológico da cidade foi mantido durante e após o evento de massa, para identificar possíveis alterações associadas, e incluiu a análise dos dados da Vigilância Sentinela, dos Sistemas de Informação em Saúde e dos atendimentos das unidades de saúde da rede pública municipal, divulgados nos painéis epidemiológicos do Centro de Inteligência Epidemiológica ^14^. A Vigilância Sentinela é um modelo de vigilância com coleta semanal de amostras laboratoriais para o monitoramento de arboviroses, síndromes gripais, doenças diarreicas agudas e conjuntivite, em estabelecimentos de saúde estratégicos, visando à detecção precoce de agentes causadores dessas doenças ^9^. O consolidado do monitoramento realizado no pós-evento foi encaminhado semanalmente, por meio de informe técnico, às instâncias de saúde estadual e federal.

## Resultados 

No pré-evento, a análise do cenário epidemiológico realizada diariamente nas quatro semanas antecedentes possibilitou identificar: o aumento de casos de coqueluche nos cenários local, nacional e internacional; um caso importado de sarampo no cenário nacional; surtos de doença diarreica aguda no cenário nacional; casos de Oropouche no cenário nacional (região extra-amazônica) e internacional; e casos humanos de gripe aviária após exposição laboral no cenário internacional. Com base nesses dados, foi elaborado um guia rápido com orientações sobre sarampo, varicela, mpox, arboviroses e coqueluche, para auxiliar os trabalhadores dos postos de saúde do evento na identificação de casos e na diferenciação de casos de doenças exantemáticas e mpox. Também foi estabelecido um fluxo para encaminhamento de casos suspeitos dessas doenças à unidade de saúde de urgência e emergência mais próxima ao local do evento, visando oportunizar a coleta de amostras para confirmação laboratorial.

Em todos os dias do evento, inicialmente foi realizada visita aos sete postos de saúde para apresentação da equipe de vigilância e orientações sobre doenças e agravos de notificação compulsória, eventos e emergências em saúde pública. Posteriormente, rondas foram realizadas a cada três horas nos postos de saúde para avaliação dos boletins de atendimento e busca ativa de casos de doenças, agravos e eventos de notificação compulsória. Foram realizadas 135 rondas. Ao final de cada ronda, os dados de atendimentos, notificações e remoções foram repassados à gestão municipal da vigilância em saúde. Ao final de cada dia, o consolidado das informações foi encaminhado à vigilância em saúde local, à Secretaria de Estado de Saúde do Rio de Janeiro e ao Ministério da Saúde. 

Do total de atendimentos realizados nos postos de saúde do evento (n=8.835), 2,7% (n=243) foram de doenças e agravos de notificação compulsória, dos quais a maioria foi de síndrome gripal (42,0%) e intoxicação exógena (32,9%). Foram identificados e notificados três surtos, um de doença diarreica aguda e dois de intoxicação exógena por spray de pimenta, o que oportunizou a investigação epidemiológica e implementação de medidas de controle in loco (Tabela 1).

**Tabela 1:** Frequências absolutas (n) e relativas (%) das doenças, agravos e eventos em saúde pública notificados no festival de música. Município do Rio de Janeiro, setembro de 2024 (n=243)

**Doenças/agravos/eventos**	**n (%)**
Síndrome gripal	102 (42,0)
Intoxicação exógena	80 (32,9)
Acidente de trabalho	45 (18,5)
Acidente por animal peçonhento	9 (3,7)
Violência interpessoal	2 (0,8)
Dengue	2 (0,8)
Surto de intoxicação exógena	2 (0,8)

No pós-evento, foi realizado o monitoramento do cenário epidemiológico local durante as quatro semanas posteriores ao evento. Não foram identificadas mudanças no perfil epidemiológico da cidade do Rio de Janeiro. Os resultados obtidos foram encaminhados semanalmente à Secretaria de Estado de Saúde do Rio de Janeiro e ao Ministério da Saúde, por meio de informes técnicos. 

Para avaliar a atuação no evento de massa, realizou-se reunião técnica com a equipe do CIEVS-Rio e a Superintendência de Vigilância em Saúde, com o objetivo de refletir sobre o método utilizado e discutir pontos a serem aprimorados. Como desdobramento, definiu-se que as estratégias a seguir serão implementadas em eventos futuros. 

Monitoramento dos níveis de calor na cidade do Rio de Janeiro antes e durante os eventos de massa: considerando o atual contexto das mudanças climáticas e a frequência crescente de episódios de calor extremo no município do Rio de Janeiro, a prefeitura instituiu a classificação dos níveis de calor com base em métricas que combinam temperatura, umidade relativa do ar e tempo de exposição ^15^. O monitoramento desses níveis possibilitará o planejamento e a implementação de estratégias voltadas à mitigação dos efeitos adversos do calor sobre a saúde da população e dos trabalhadores, especialmente durante a realização de eventos de massa.

Monitoramento dos atendimentos relacionados ao evento de massa na Rede de Urgência e Emergência: serão analisados dados dos prontuários eletrônicos da Rede de Urgência e Emergência por meio dos painéis do Centro de Inteligência Epidemiológica ^14^. A busca será realizada a partir de descritores específicos relacionados ao evento, o que possibilita a identificação de casos que tenham buscado atendimento em equipamentos de saúde externos.

## Discussão 

O objetivo deste estudo foi descrever a estratégia de vigilância em saúde implementada pelo CIEVS-Rio em eventos de massa realizados na cidade do Rio de Janeiro, destacando a experiência em um festival de música. A estratégia descrita envolveu atividades de vigilância ativa e vigilância baseada em eventos. A identificação prévia de riscos epidemiológicos, como o aumento de casos de coqueluche e sarampo, possibilitou a elaboração de materiais educativos e a organização de fluxos para encaminhamento de casos suspeitos atendidos no evento para unidades municipais de saúde. A vigilância ativa realizada durante o evento possibilitou a detecção oportuna de doenças e agravos de notificação compulsória, além da identificação e resposta imediata a surtos. No período posterior ao evento, o monitoramento contínuo do cenário epidemiológico não indicou alterações no padrão de ocorrência de doenças na cidade. A experiência gerou aprendizados que serão incorporados na preparação de eventos futuros.

Este relato de experiência apresentou algumas limitações que devem ser consideradas. Por se tratar da descrição de estratégia de vigilância em saúde desenvolvida para um contexto urbano específico, os métodos empregados podem não ser replicáveis em outras localidades, especialmente naquelas com menor infraestrutura de saúde ou diferentes características socioambientais. A análise dos dados de saúde foi centrada nos atendimentos realizados nos postos de saúde durante o evento, o que pode não refletir completamente a totalidade dos casos relacionados, visto que há a possibilidade de o público e os trabalhadores procurarem atendimento em unidades de saúde externas.

O planejamento e a resposta à disseminação de doenças transmissíveis e a ocorrência de eventos e emergências de saúde pública, decorrentes do intenso fluxo nacional e internacional de pessoas, também foram discutidos em outras publicações ^3^ ^,^ ^4^ ^,^ ^5^ ^,^ ^6^ ^,^ ^16^ ^,^ ^17^ ^,^ ^20^. Similarmente à estratégia de vigilância em saúde da cidade do Rio de Janeiro apresentada neste estudo, foram apontadas ações de prevenção e mitigação dos riscos à saúde pública em eventos de massa, com destaque para o monitoramento epidemiológico, a disponibilização de serviços de saúde no local do evento e a elaboração de protocolos de resposta rápida ^17^ ^,^ ^20^. No entanto, até onde se pode observar, não foram identificados estudos que sistematizam a atuação da vigilância em saúde em eventos de massa considerando as etapas antes, durante e após os eventos, o que confere inovação a esta abordagem. 

Apesar de a atuação relacionada às doenças transmissíveis ser principalmente referida na literatura sobre eventos de massa, fatores como riscos relacionados a acidentes, eventos climáticos extremos e infraestrutura inadequada podem resultar em fatalidades e desastres durante grandes eventos e, portanto, também devem ser considerados ^18^. A avaliação da infraestrutura local e de riscos ambientais realizadas pela vigilância em saúde da cidade do Rio de Janeiro na fase pré-evento estão em acordo com essas recomendações. O monitoramento das condições climáticas e suas consequências para a saúde do público presente nos eventos de massa é uma estratégia que será implementada em futuros eventos de massa, como desdobramento das “lições aprendidas” no festival de música descrito neste relato. 

A alta concentração de pessoas, as condições ambientais adversas e a possibilidade de ocorrência de doenças transmissíveis são fatores que demandam planejamento estratégico e ações intersetoriais coordenadas. Nesse sentido, torna-se cada vez mais relevante a implementação de sistemas de vigilância sensíveis e responsivos, capazes de captar sinais precoces de agravos à saúde e viabilizar respostas rápidas e integradas ^5^ ^,^ ^17^. A introdução de tecnologias para os sistemas de vigilância tem contribuído significativamente nesse cenário, ao possibilitar a coleta de dados em tempo real e a comunicação eficiente entre as equipes, o que permite intervenções mais oportunas e precisas. Embora na cidade do Rio de Janeiro ainda não tenha sido desenvolvido um sistema de informação específico para o monitoramento epidemiológico durante os eventos de massa, a busca ativa de casos de doenças e agravos de notificação compulsória cumpre esse papel. Além disso, a futura utilização de dados dos painéis epidemiológicos do Centro de Inteligência Epidemiológica possibilitará monitorar os casos de doenças e agravos de interesse para além do espaço físico do evento.

As principais doenças e agravos de notificação compulsória notificados durante o evento foram intoxicação exógena, síndrome gripal e acidente de trabalho. O consumo de álcool e drogas foi destacado como importante motivo de atendimento de saúde em eventos de massa, o que promoveu aumento significativo nos casos de intoxicação, além de lesões relacionadas a traumas e acidentes ^17^ ^,^ ^18^. Esses resultados corroboram com os obtidos neste estudo, pois a maioria dos casos de intoxicação exógena identificados foi decorrente do consumo abusivo de álcool e drogas. 

As notificações de síndrome gripal podem ser atribuídas, em parte, à sazonalidade dos vírus respiratórios no Brasil, como o da influenza e o vírus sincicial respiratório que têm maior circulação em determinadas épocas do ano, como no outono e no inverno ^19^. Essa sazonalidade contribui para a maior incidência das doenças respiratórias, especialmente em locais de grande aglomeração, como festivais de música, onde a proximidade entre as pessoas favorece a transmissão de vírus respiratórios.

A atuação do CIEVS-Rio em eventos de massa envolve um conjunto integrado de ações antes, durante e após o evento, essenciais para a identificação precoce de riscos e resposta rápida a situações que podem constituir emergências para a saúde pública. Essas medidas visam mitigar riscos e garantir a segurança sanitária em cenários de grande aglomeração de pessoas, onde a diversidade de origens dos participantes pode intensificar os desafios epidemiológicos. No festival de música, as ações implementadas oportunizaram a preparação adequada, a detecção precoce e a resposta rápida a potenciais emergências em saúde pública. As experiências adquiridas auxiliarão no planejamento de futuros eventos. 

O compartilhamento das estratégias e experiências do CIEVS-Rio poderá contribuir para o aprimoramento contínuo das práticas de vigilância em saúde em eventos de massa, fortalecendo a capacidade de resposta de diferentes esferas de gestão do Sistema Único de Saúde que possuam desafios sanitários semelhantes. A troca de experiências entre municípios e o fortalecimento das redes de vigilância configuram-se como estratégias cruciais para o aprimoramento contínuo das práticas de vigilância em saúde em eventos de massa. Essas ações, além de potencializar a capacidade de resposta local, contribuem significativamente para a capacitação das equipes técnicas, promovendo a atuação mais eficiente e integrada.
